# Implementing and evaluating online advance care planning training in UK nursing homes during COVID-19: findings from the Necessary Discussions multi-site case study project

**DOI:** 10.1186/s12877-022-03099-z

**Published:** 2022-05-13

**Authors:** Emily Cousins, Nancy Preston, Julie Doherty, Sandra Varey, Andrew Harding, Adrienne McCann, Karen Harrison Dening, Anne Finucane, Gillian Carter, Gary Mitchell, Kevin Brazil

**Affiliations:** 1 Division of Health Research, Faculty of Health and Medicine, Lancaster University, Lancaster, UK; 2 School of Nursing and Midwifery, Queen’s University Belfast, Belfast, UK; 3 Innovation Value Institute, Maynooth University, Maynooth & Age Friendly Ireland, Ireland; 4 School of Nursing and Midwifery, Faculty of Health and Life Sciences, De Montfort University, Leicester & Dementia UK, London, UK; 5 Clinical Psychology, University of Edinburgh, Edinburgh & Marie Curie Hospice Edinburgh, Edinburgh, UK

**Keywords:** COVID-19, Advance care planning, Nursing homes, Training, Online

## Abstract

**Background:**

Advance care planning in nursing homes is important to ensure the wishes and preferences of residents are recorded, especially during the COVID-19 pandemic. However, care staff and family members frequently report feeling unprepared for these conversations. More resources are needed to support them with these necessary discussions. This research aimed to develop, implement and evaluate a website intervention for care staff and family members to provide training and information about advance care planning during COVID-19.

**Methods:**

The research was a primarily qualitative case study design, comprising multiple UK nursing home cases. Data collection included semi-structured interviews with care staff and family members which were coded and analysed thematically. A narrative synthesis was produced for each case, culminating in a thematic cross-case analysis of the total findings. Theoretical propositions were refined throughout the research.

**Results:**

Eight nursing homes took part in the study, involving 35 care staff and 19 family members. Findings were reported according to the RE-AIM framework which identified the reach, effectiveness, adoption, implementation and maintenance of the intervention. Themes included: website content that was well received; suggestions for improvement; implementation barriers and facilitators; examples of organisational and personal impact.

**Conclusions:**

Four theoretical propositions relating to advance care planning in nursing homes are presented, relating to: training and information needs, accessibility, context, and encouraging conversations. Implications for practice and training include an awareness of diverse learning styles, re-enforcing the right to be involved in advance care planning and encouraging opportunities for facilitated discussion.

**Trial registration:**

ISRCTN registry (ID 18003630) on 19.05.21.

**Supplementary Information:**

The online version contains supplementary material available at 10.1186/s12877-022-03099-z.

## Background

The significant impact of the COVID-19 pandemic in UK nursing homes has been well documented [1, 2]. Residents have an increased susceptibility to COVID-19, due to multi-morbidities and frailty. Consequently, the pandemic has emphasised the importance of advance care planning for this population. Advance care planning allows adults to understand and share their personal values and preferences regarding future care [3]. Advance care planning is relevant to everyone, irrespective of age or health status, to ensure that people’s care wishes are clearly documented ahead of time. Having these conversations in advance of ill health or ageing ensures individuals can communicate their wishes while they are still able to. Advance care plans vary, but they are likely to include decision-making relating to certain medical treatments or arrangements for care at the end of life [3].

Advance care planning is critical for nursing home residents and their families, and its importance was heightened during the pandemic [4]. The health of those who contract COVID-19 can change rapidly, therefore it is vital to know their care preferences in case they are unable to contribute to shared decision-making conversations. The circumstances of COVID-19 therefore necessitated a proactive approach to advance care planning [1, 2, 5]. However, it is also well documented that care staff and family members find these conversations challenging [3, 6].

Consequently, the Necessary Discussions project aimed to produce and evaluate a training and information website (the intervention) to support care staff and family members to talk about advance care planning during a COVID-19 outbreak. As stated in the study protocol [7], the aim of the intervention was to provide care staff and family members with accessible information about advance care planning during COVID-19, including practical details of how to conduct conversations about future care wishes for a relative in a nursing home.

The rationale for the intervention was a belief that providing care staff and family members with relevant knowledge would encourage more advance care planning discussions during a COVID-19 outbreak. Nursing home residents were not directly involved in the intervention due to COVID-19 restrictions. However, the rights and needs of the resident, and promotion of their active involvement in advance care planning wherever possible, were strongly advocated throughout the intervention. Moreover, the active involvement of care staff and family members in advance care planning is likely to increase positive care outcomes for residents [3, 6, 7].

Research identified a lack of web-based resources for the public about advance care planning during COVID-19 [5] – a gap this study responded to. As the clinical response was in flux during the early months of the pandemic, the intervention also aimed to display a synthesis of expert guidance that was produced concurrently relating to advance care planning in nursing homes during COVID-19.

The intervention was implemented with care staff and family members. The research was conducted using a primarily qualitative case study design. This paper reports findings from the intervention evaluation, which aimed to understand:

- barriers and facilitators to implementing the intervention;
- feedback regarding the content and information of the intervention;
- perceived impact of the intervention in relation to knowledge and changes to practice.

### Development of the intervention

The intervention (website) was developed as follows:

1. A rapid review and synthesis of COVID-19 related UK guidance about advance care planning informed the intervention’s content. Detailed methods and findings from the rapid review will be published separately. The intervention contained two distinct areas: a training programme for care staff, comprised of units and learning objectives, and an information section for family members (Table 1). The family member resource, deliberately not labelled as training in an attempt to make it more accessible, was aimed at those with a relative or close friend resident in a nursing home. The intervention sought to provide an overview of advance care planning during COVID-19, and included tips and guidance for staff and family members. It was envisaged that participants could complete the intervention within 2 h, across multiple, shorter sessions if necessary.
2. Researchers worked with an Expert Reference Group (ERG) to finesse the intervention’s content to ensure accuracy, meeting three times throughout the project. The ERG (*n* = 14) included UK based clinicians, academics, practitioners, care providers and family members. ERG members provided feedback on the information presented in the intervention to ensure it represented best practice in relation to advance care planning and provided a comprehensive summary of the most important elements. ERG members also gave strategic advice for engaging with nursing homes during the project and suggestions for disseminating research findings effectively amongst practitioners.
3. Concurrently, researchers worked with an integrated communications company to develop the intervention’s design and layout, to optimise information clarity and accessibility (see additional file 1). This included the filming and production of short videos, featuring academics and practitioners, to accompany the website text.
4. Following implementation and evaluation of the intervention, a round of revisions were made to the intervention based on participant feedback.

**Table 1:** Summary of the care staff and family member sections of the intervention

Care staff training	Family member information
Unit 1: Introduction to advance care planning in the context of a COVID-19 outbreak	1. What is advance care planning?
Unit 2: Advance care planning in the context of a COVID-19 outbreak	2. Why is advance care planning important during COVID-19?
Unit 3: How to complete an Advance Care Plan during a COVID-19 outbreak	3. What might be included in an advance care plan during COVID-19?
Unit 4: Recording and sharing Advance Care Plans during a COVID-19 outbreak	4. Who takes part in advance care planning during COVID-19?
Unit 5: Finding the words: Tips for having necessary discussions	5. How do I take part in advance care planning during COVID-19?
Unit 6: Caring for yourself during a COVID-19 outbreak	6. How do I care for myself during COVID-19?
Resources	Resources

### Evaluation methods

**Fig. 1:**
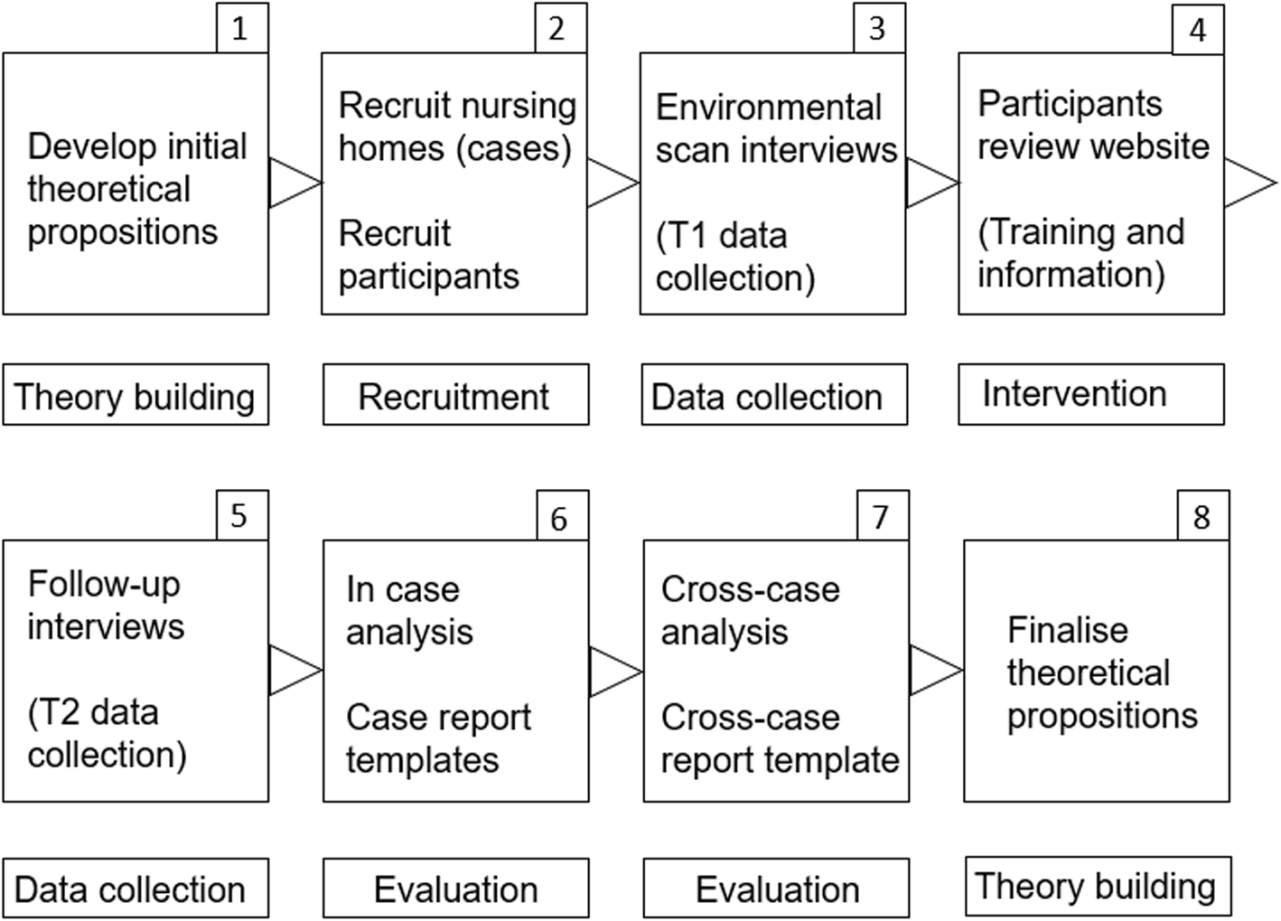
Necessary Discussions study design, implementation and evaluation

The intervention was evaluated using case study methodology (Fig. 1), using approaches outlined by Yin [8, 9] and Gillham [10], alongside other applications of case study methodology in healthcare fields [11–13]. This project took a primarily qualitative approach to case study, utilising participant interviews to map context and impact at an organisational and individual level. This allowed a depth of understanding to develop, as proven by previous case study research focussed on intervention implementation [14] and the evaluation of complex healthcare interventions [11]. A pre-post evaluation method was not used because the aim of the evaluation was to collect initial, qualitative feedback about the participants’ impressions of the intervention. Future evaluations of the intervention could consider this design. The theoretical RE-AIM framework (Reach, Effectiveness, Adoption, Implementation, Maintenance) [15, 16], which guides the implementation of healthcare interventions to maximise efficacy will be used to articulate and evaluate the research findings in order to demonstrate impact.

### Theoretical prepositions

Case study research is guided by hypothesis statements, known as theoretical propositions. These are developed at the start of the research and re-visited during data analysis, to compare expectations with findings [8, 9]. They are guided by the objectives of the research project, in this instance the aim to provide care staff and family members with accessible information about advance care planning in order to encourage discussions about future care wishes. The theoretical propositions for this study (Table 2) were devised by the project team through a series of meetings and discussions, and were based on the academic evidence-base, the rapid-review and synthesis of COVID-19 literature and tacit knowledge.

**Table 2:** Theoretical propositions guiding the Necessary Discussions project

Proposition theme	Propositions for care staff	Propositions for family members
1. Training needs	Care staff require specific training about advance care planning during a COVID-19 outbreak	Family members require specific information about advance care planning during a COVID-19 outbreak
2. Online accessibility	Care staff are able to access training online	Family members are able to access information resources online
3. Technology acceptance	Care staff will find an online training website useful and easy to use	Family members will find an online information website useful and easy to use
4. Barriers and facilitators	There are barriers and facilitators to providing care staff with online training about advance care planning during a COVID-19 outbreak	There are barriers and facilitators to providing family members with online resources about advance care planning during a COVID-19 outbreak
5. Encourage conversations	Providing training online to care staff will encourage conversations about advance care planning during a COVID-19 outbreak	Providing information online to family members will encourage conversations about advance care planning during a COVID-19 outbreak
6. Improve knowledge	Completing online training will improve care staff knowledge about advance care planning during a COVID-19 outbreak	Reviewing online information resources will improve family member knowledge about advance care planning during a COVID-19 outbreak
7. Increase confidence	Providing training online to care staff will increase confidence about advance care planning during a COVID-19 outbreak	Providing information online to family members will increase confidence about advance care planning during a COVID-19 outbreak

### Multiple case design and case boundaries

Nursing homes across Northern Ireland (N.I) (*n* = 3), England (*n* = 3) and Scotland (*n* = 2) were recruited to the study. In each home, care staff and family members were recruited to use the intervention for training or accessing information. Consequently, this was a multiple case design, where each nursing home functioned as an individual ‘case’ (unit of analysis) [8]. The scope of each case was the nursing home itself. This provided an organisational boundary to each case, containing all of the participants recruited from that respective nursing home. This enabled intervention analysis at an individual nursing home level, including the contextual significance of each setting, as well as synthesising findings across all cases to identify data patterns and draw generalisations [12].

### Case and participant selection

Due to the pandemic, participating nursing homes represented a convenience sample. Each recruited home was registered to deliver nursing and personal care to its residents. The study focussed on nursing homes, rather than residential homes more broadly, so that nursing staff *and* care staff could participate in the intervention. Efforts were made to ensure the participating nursing homes represented diverse characteristics, such as location, size and type of care provided. For example, the research team met frequently to discuss recruitment and consequently targeted different types of homes within and between countries. Due to COVID-19 restrictions, it was not possible to visit the nursing homes or participants during the research. The implications of this are discussed later.

Care staff were recruited based on having a resident or family facing role, which might require them to initiate or field conversations about advance care planning during the COVID-19 pandemic. This included registered nurses and managers, care assistants, activity co-ordinators and administrative staff. Family members were recruited using the following inclusion criteria: 18 years or older; actively involved in the resident’s care; able to understand written and spoken English; and with access to a digital device e.g. mobile phone, tablet or computer. Family members were not required to have any previous experience of advance care planning in order to be recruited to the study. The health status or age of their relative in the nursing home did not form part of the inclusion or exclusion criteria as all individuals can benefit from advance care planning.

All eligible participants were identified by the nursing home manager or care staff as relevant. They were recruited remotely due to COVID-19 restrictions, using an information pack (sent via post or email) containing a Participant Information Sheet (PIS) and consent form. Signed or verbally recorded informed consent was obtained for each participant. Further participant recruitment details are outlined in the study protocol [7]. Ethical approval was obtained for the study (Health and Social Care Research Ethics Committee B (HSC REC B—20/NI/0173)).

### Within case data collection, data analysis and case reports

#### Data collection

Data collection took place at two time points. Time point 1, the environmental scan, took place with the nursing home manager, care staff and family members in each participating home. Participants completed a semi-structured interview via phone with a member of the research team, lasting approximately 30 min. Questions included: “*Have you experience of developing or updating advance care plans since the beginning of the COVID-19 pandemic?*”; “*How do family members currently participate in the development of advance care plans?*”; “*What would make it easier for you to complete the training?*” The rationale for these questions was to provide insights into each nursing home’s implementation context, their current practices relating to advance care planning during COVID-19 and any perceived barriers or facilitators regarding the intervention’s implementation. Consequently, the environmental scan established a contextual baseline at a case level, and at an individual participant level, which informed subsequent data analysis and reporting. Following interviews, the researchers recorded key observations in narrative field notes, for example insights relating specifically to the theoretical propositions. Additionally, each nursing home was asked to complete a profile questionnaire to provide further contextual detail.

Following completion of the environmental scan interviews, participants were given access to the intervention. Care staff were given an individual log-in to the training part of the website, to monitor access. Nursing homes were provided with computer tablets so that staff could access the website during working hours. Family members were emailed a link to the website and were given a content warning in case the material was upsetting. Furthermore, the website contained contact details of organisations that could provide support, and a distress protocol was in place throughout the study to address any emotional consequences resulting from family members accessing the information. On a few occasions, to maximise participant numbers due to tight project time scales, participants proceeded to the intervention without an environmental scan interview. In this instance individuals were given a full verbal briefing about the project and an opportunity to ask any questions.

Following the intervention, participants took part in a follow-up semi-structured interview (Time point 2). The Technology Acceptance Model (TAM), a tool frequently used to evaluate e-learning, was employed to develop the Time point 2 interview schedule, to identify the usability, usefulness and impact of the website [17]. Questions included: “*Were there any benefits to completing this training?*”; “*Have you applied this training in practice*?”; “*Were you comfortable looking at the information on the internet?*”. The rationale for these questions was to elicit feedback about the intervention, for example relating to its accessibility, and to understand its impact at a personal and organisational level.

#### Data analysis

Each interview was anonymised, transcribed and coded. Coding was undertaken inductively (data led) by the researcher responsible for that particular case. A coding table was used to record codes in relation to each interview question, underpinned by data excerpts that illustrated each one. Codes were reflexively analysed, then grouped into themes to generate salient findings for each case [18]. During coding and analysis, the researchers discussed interpretations of the data to enhance methodological rigour [19]. The field notes contributed to these discussions and interpretations where relevant during data analysis, and helped to triangulate the research findings to develop a deeper understanding of the data, but they were not formally coded. Quantitative data, namely participants’ previous experience of advance care planning, were analysed using descriptive statistics. Data were password protected and stored securely using Microsoft Teams folders, according to the permissions set out in the study’s ethical approval.

#### Case reports

Following data analysis, a case report was completed by the relevant researcher which provided a narrative synthesis of each case (nursing home), including relevant quotations from the interview data, profile questionnaire and researcher field notes. The case report template was developed by the researchers prior to data analysis and comprised four sections, corresponding to the aims of the intervention evaluation: 1) nursing home environment (context); 2) implementation of training (barriers and facilitators); 3) content of training; 4) perceived impact of training (knowledge and changes to practice).

### Cross-case analysis

Following completion of each individual case report, researchers met to complete the cross-case analysis. The aim was to conduct a thematic synthesis of the entire study’s data, to identify patterns and generalisations across the data sets, resulting in a conclusive evaluation of the intervention [20]. A cross-case analysis template was produced prior to the exercise, and comprised the same sections as before. Discussing each case in turn, the cross-case template was populated with findings from each case, which resulted in definitive, high-level themes that identified similarities across all data sets.

The cross-case analysis, and resulting intervention evaluation, was also guided by the RE-AIM framework [15, 16], which can be considered as follows:

- Reach (proportion of participants who accessed the intervention);
- Effectiveness (impact of the intervention on a personal level e.g. knowledge);
- Adoption (participants’ acceptability of the intervention e.g. the website content);
- Implementation (barriers and facilitators to completing the intervention);
- Maintenance (impact of the intervention on an organisational level e.g. changes to practice).

## Results

Table 3 presents a summary of findings from the cross-case analysis relating to effectiveness, adoption, implementation and maintenance. Each component of the RE-AIM framework is discussed in detail below.

**Table 3:** A summary of thematic findings from the cross-case analysis

	**Care staff**	**Family members**
**Effectiveness**	1) Increased awareness and understanding of advance care planning	1) Increased knowledge and understanding of advance care planning
	2) An opportunity to build on existing skills	2) Reassurance about advance care planning
	3) Increased confidence for advance care planning	3) Permission to be involved in advance care planning
	4) Preparedness for advance care planning conversations with families	4) Confidence and empowerment about advance care planning
	5) Increased willingness to talk about advance care planning	5) Feeling involved and valued as a care partner
**Adoption**	**Content that was well received**	
	1) A comprehensive overview of advance care planning	1) Key points of advance care planning explained
	2) Information about self-care	2) Support resources
	3) Audio-visual website design	3) Vibrant website design
	4) Inclusion of different perspectives and voices	4) Gentle tone
	5) Appropriate language and tone	5) Videos
	**Suggestions for improvement**	
	1) Facilitated blended learning	1) Supplementary printed information
	2) Assessment of learning	2) Clarify COVID-19 focus
	3) Evidence of training	3) Clarify legal aspects
	4) Advanced training options	
	5) Real life examples	
**Implementation**	**Barriers**	
	1) Computer skills	1) Emotional content
	2) Time	2) No access to technology
	**Facilitators**	
	1) Ensuring the website is easy to use	1) Simple information
	2) Working in groups	2) Trustworthy information
		3) Bitesize information
**Maintenance**	1) Advance care planning policies reviewed	1) Prompted conversations with relative in the nursing home
	2) Advance care planning paperwork reviewed	2) Prompted conversations with care staff
	3) Shared learning between colleagues	
	4) Desire to roll out training	

### Reach: cases and participants in the study

Eight nursing homes (cases) were recruited to the study (N.I), *n* = 3; England, *n* = 3; Scotland, *n* = 2) (Table 4). A total of 35 care staff and 19 family members participated across all cases. Care staff is used throughout the Results section as a unified term, but where contextually relevant individual staff roles are referenced, for example a nurse or manager.

**Table 4:** An overview of each case in the study

Case number	Home structure	Care provided	Size	Research participants	No. of staff participants with experience of advance care planning	No. of family member participants with experience of advance care planning
1 (N.I.)	Independent^a^	Nursing and personal care	~ 40 beds	Nurse: 2	2/4	3/3
				Care assistant: 2		
				Family member: 3		
2 (N.I.)	Independent	Nursing and personal care	~ 70 beds	Nurse: 3	3/5	3/3
				Care assistant: 2		
				Family member: 3		
3 (N.I)	Local private provider ^a^	Nursing and personal care	~ 90 beds	Manager: 1	1/3	1/1
				Nurse: 2		
				Family member: 1		
4 (England)	Local private provider	Nursing and personal care	~ 50 beds	Manager: 2	4/6	3/3
				Nurse: 1		
				Care assistant: 1		
				Administrator: 1		
				Activity co-ordinator: 1		
				Family member: 3		
5 (England)	Local private provider	Nursing and personal care	~ 50 beds	Manager: 2	5/6	-
				Nurse: 1		
				Senior carer: 3		
				Family member: 0		
6 (England)	Local private provider	Nursing and personal care	~ 50 beds	Manager: 1	3/3	5/5
				Nurse manager: 2		
				Family member: 5		
7 (Scotland)	Independent	Nursing and personal care	~ 40 beds	Nurse: 1	1/4	0/1
				Advance care practitioner: 1		
				Care assistant: 2		
				Family member: 1		
8 (Scotland)	Local private provider	Nursing and personal care	~ 30 beds	Nurse: 2	2/4	3/3
				Care assistant: 2		
				Family member: 3		
**Total**				**Staff: 35**	**21**	**18**
				**Family members: 19**		

^a^Independent and local private provider denote nursing homes which are privately owned, as opposed to nursing homes which are run by the voluntary and public sectors. Independent homes are not part of a chain, whereas local private providers own several homes within the region

Participant recruitment was fairly consistent across the cases, namely that a similar number of participants were recruited for each case, and the resulting numbers provided significant insights for qualitative findings [14]. It proved easier to recruit staff than family members, perhaps due to availability and staff acting as gatekeepers. Attrition rates during the study were favourable, and minimal numbers of participants were lost to follow-up (care staff, *n* = 5; family members, *n* = 1).

All but one family member had prior experience of advance care planning and being involved in discussions relating to their relative:

*“I’ve always been involved with staff at the care home”* (England_1, family member 3).

Two thirds of the staff participating had experience of hosting advance care planning conversations with relatives, but for some this was a new area:

*“I don’t really know what it involves or what it’s really about”* (N.I_1, care staff 1).

Table 4 presents this quantitative data, including participants’ previous experience of advance care planning.

### Effectiveness: impact of the intervention on a personal level

The cross-case analysis identified several themes expressing what care staff gained from completing the training: 1) increased awareness and understanding of advance care planning; 2) an opportunity to build on existing skills; 3) increased confidence for advance care planning; 4) preparedness for advance care planning conversations with families; 5) increased willingness to talk about advance care planning.

Some care staff made the connection between enhanced understanding and increased confidence:

“*It’s gaining knowledge in something I didn’t have yet, which has now helped me in work. A lot more confident with care plans and everything else.”* (Scotland_2, care staff 2).

Others demonstrated an enthusiasm for having an opportunity to develop their skills and role:

*“It would enhance my skills…and my knowledge and I can apply it to my workplace as well.”* (N.I_3, care staff 2).

Even staff who had experience of undertaking advance care planning felt the training was valuable as a prompt:

“*I think it’s definitely very good refresher training…I haven’t had any advance care planning training since the start of COVID*” (Scotland_1, Nurse 1).

There were several striking examples of staff demonstrating an increased confidence, willingness and sense of permission to engage with advance care planning conversations:

“*It’s given me the confidence to think, yes, this is part of my role…to have this ongoing discussion…The training has given me permission to implement it across the board*.” (England_1, care staff 5).

“*I have a lady downstairs now…she doesn’t have a DNAR in place. [Before the training] we had a conversation and we found it really, really hard…But after we did this [the training], we *broached* it in a different format…and do you know, they are going to put a DNAR in place…it made it quite nice, really, having the conversation with them. So yeah, it was very worthwhile*” (England_3, nursing home manager).

For family members, the following themes were identified in relation to what they gained from the intervention: 1) increased knowledge and understanding of advance care planning; 2) reassurance about advance care planning; 3) permission to be involved in advance care planning; 4) confidence and empowerment about advance care planning; 5) feeling involved and valued as a care partner.

Family member feedback consistently stated the intervention provided a helpful overview of advance care planning:

*“It was very comprehensive, it’s very clear and yeah, it’s very relevant.”* (Scotland_2, family member 2).

Others responded with how the intervention made them feel, recognising that the provision of information made them feel valued and recognised as a carer:

“*It’s the first thing in all the ten years I’ve been involved with the care home…that wants to involve relatives with what’s happening to their resident*” (England_1, family member 1).

This family member echoed others by stating that the information had reinforced her right to be involved in advance care planning; the sense of empowerment is palpable:

“*It’s made me realise…I have the right to be involved…I can say, I want to be involved. I want to be made aware…on a regular basis of any decisions that are made.”* (England_1, family member 3).

### Adoption: acceptability of the intervention content

The cross-case analysis identified themes relating to training content that was well received by care staff: 1) a comprehensive overview of advance care planning; 2) information about self-care; 3) audio-visual website design; 4) inclusion of different perspectives and voices; 5) appropriate language and tone.

Staff appreciated the professionally produced videos communicating advice and information about advance care planning from academics and practitioners:

*“I loved the videos. I thought they were spot on. I didn’t get bored watching them.”* (N.I_1, nursing home manager).

Moreover, staff appreciated that the intervention provided a variety of perspectives relating to advance care planning, explaining the process from the position of care providers, families and residents:

“*It was all different kinds of voices and all different kinds of enthusiasm as well*.” (England_1, care staff 4).

Staff responded especially positively to the information about self-care following the exhaustion and stress of the pandemic, which gave them permission to consider their own needs when caring for others:

“*I really liked as well the part about self-care during the COVID…where you suggest a couple of ways how to look after ourselves…I never thought about this*.” (England_3, Nurse 1).

For family members, the following themes illustrate aspects of the information that were viewed positively: 1) key points of advance care planning explained; 2) support resources; 3) vibrant website design; 4) gentle tone; 5) videos.

Consistently, family members praised the eye-catching design of the intervention which presented the information with clarity:

“*I thought it was easy to access and the colours were…nice, vibrant*” (N.I_2, family member 1).

In particular, family members appreciated the resources section, which presented key useful documents all in one place:

“*I actually really felt that the resources section is so valuable…some of that information is just really good*” (Scotland_2, family member 2).

No negative feedback was recorded from care staff. The following themes describe suggestions that were made to improve the content: 1) facilitated blended learning; 2) assessment of learning; 3) evidence of training; 4) advanced training options; 5) real life examples.

Care staff recognised that having an opportunity to discuss the intervention training with colleagues, for example how to apply it to the particular circumstances of their own nursing home, would help to strengthen and embed the learning:

“*Maybe if there was actually another professional that came in as well and sat down and went through some of it.”* (N.I_1, care staff 2).

Others suggested a short assessment or quiz would help to clarify what they had learned:

*“Maybe a couple of even multi choice questions or something at the end, to see if I had picked it up and understood everything”* (N.I_2, care staff 3).

Family members gave suggestions for improving the information that was provided, which could be categorised into three broad themes: 1) supplementary printed information; 2) clarify COVID-19 focus; 3) clarify legal aspects.

Family members requested clarity around the COVID focus of the intervention, in particular confirming that the intervention was relevant to everyone with a family member residing in a nursing home:

“*I think it could be misleading that it only really applies to people suffering from COVID or has concerns around COVID, rather than generally looking at end of life advance care planning [during COVID]”* (England_3, family member 3).

One family member suggested that the intervention could be more age inclusive by stating explicitly that advance care planning is important for adults of all ages who are resident in a nursing home.

Others requested more information about legal aspects that were mentioned in the information, particularly those who had gone through the process of putting in place lasting power of attorney:

*“I think what I would have liked was perhaps a little bit more on what the legal options are*” (Scotland_2, family member 3).

### Implementation: barriers and facilitators to completing the intervention

The cross-case analysis identified barriers (perceived and actual) that may prevent care staff from completing the training: 1) computer skills; 2) time.

Overwhelmingly, care staff reported that having time to complete the training was the most significant barrier they faced:

*“Probably time…if I was on shift today and there was an online training course that had to be completed today, there would be no chance that I would be getting near it within my twelve-hour shift.”* (N.I_1, care staff 1).

In practice, staff did manage to find the time to complete the training. However, it was evident some staff had been able to take longer to engage with the materials than others, suggesting that time was still a significant factor relating to implementation. Moreover, several staff reported they had completed the training outside of their working hours.

Some participants recognised that computer skills may be a barrier:

“*Somebody would have a problem with the computer skills, they might find it confusing. So logging in and finishing, moving between the units.”* (England_3, Nurse 1).

There were no significantly common themes identified by family members relating to barriers that would prevent them from viewing the information on the website. One suggested the content might be emotional, while another recognised that not having access to technology may be a barrier:

“*Not everybody has access…you always have to think about everyone who uses the service and making sure everybody gets the information…make sure that they are not forgotten about.”* (N.I_2, family member 1).

Two themes were identified relating to facilitators (perceived and actual) that may support care staff to complete the online training: 1) ensuring the website is easy to use; 2) working in groups.

A majority of care staff reported that they found the intervention well laid out and easily accessible:

*“Well it was really user friendly…I loved how it was in sections”* (England_1, care staff 6).

Some staff suggested that it may be easier for them to complete the training if they worked together:

“*If it’s a small group in the workplace…you tend to see better results…it’s the reflection and the chatting about it as well, I think helps people.”* (Scotland_2, Nurse 1).

Family members identified features which would help to make the intervention as accessible as possible: 1) simple information; 2) trustworthy information; 3) bitesize information.

Several family members were discerning about the healthcare information they chose to access online. A trustworthy website, endorsed by professionals with reliable information, was an important factor in determining usage:

“*I don’t *believe* everything they say on the internet. I think you should go to the people who are qualified to… and I don’t think the internet always is*” (England_1, family member 1).

Another factor to facilitate use of the intervention was ensuring the information was easily digestible and split into clear sections:

“*Bite size information to let you know what other professionals are talking about as well.”* (England_3, family member 3).

### Maintenance: impact of the intervention on an organisational level

The cross-case analysis showed that when care staff completed the training, impacts also occurred at an organisational level: 1) advance care planning policies reviewed; 2) advance care planning paperwork reviewed; 3) shared learning between colleagues; 4) desire to roll out training.

There were strong examples of tangible and lasting impacts in nursing homes, such as updating advance care planning paperwork:

“*We updated our advance care plan, our little template…with some of the ideas from the course. So that’s very good.”* (England_2, care staff 1).

Several staff reported sharing insights from the training with other colleagues who had not taken part in the research, helping the intervention to have a ripple effect across the nursing home:

*“I can discuss this with my other staff as well. We have to work collaboratively…explain to them the importance of doing the advance care planning”* (N.I_3, care staff 2).

The majority of participating homes were also keen to embed and roll out the training and information to other staff and family members:

“*In our staff training, we would really like to be able to use it… it would be great to be able to*” (England_3, nursing home manager).

For family members, reviewing the intervention led to impacts at an organisational level: 1) prompted conversations with their relative or loved one at the nursing home; 2) prompted conversations about advance care planning with care staff.

Several family members reported taking practical actions following the intervention, such as having a conversation with the nursing home:

“*I need to start  having a chat with the care home about advance care. So it did provoke thoughts for me to actually think, I need to actually take some action here.”* (England_3, family member 4).

Others felt it was important to share the intervention with their relative in the nursing home, as a prompt for discussing care wishes:

“*I had the conversation with Dad and I showed him the website. So I wouldn’t have done that *before*…But I said, look Dad, this is what we have been involved in and this is what they are doing in terms of nursing care. And he said, I think that’s really good. He said, you see our generation, we don’t talk about the hard stuff.”* (N.I_2, family member 1).

## Discussion

Following data analysis, the theoretical propositions were refined to reflect the cross-case findings [8, 9]. The finalised theoretical propositions (Table 5) outline considerations relating to implementation of the intervention.

**Table 5:** Theoretical propositions following data analysis

Proposition theme	Propositions for care staff	Propositions for family members
1. Training and information needs	Care staff require specific training about advance care planning during a COVID-19 outbreak	Family members require specific information about advance care planning during a COVID-19 outbreak
2.Training and information accessibility	Some care staff are able to access training online, though I.T. support should be available to those who need it	Some family members are able to access information resources online, but information should be made available for those without internet access
3. Training and information context	Individual and organisational contexts inform the barriers and facilitators to providing care staff with online training	Individual contexts inform the barriers and facilitators to providing family members with online information
4.Encourage conversations	Providing training online to care staff will encourage conversations about advance care planning during a COVID-19 outbreak by: • improving knowledge • increasing confidence • giving permission	Providing information online to family members will encourage conversations about advance care planning during a COVID-19 outbreak by: • improving knowledge • empowering and valuing • giving permission

### Theoretical proposition 1: training and information needs

Prior to COVID-19, there were numerous advance care planning resources for multi-disciplinary healthcare staff and family members demonstrating the training and information needs that exist [3, 21, 22]. However, gaps remained regarding advance care planning information specifically addressing the nursing home context [23]. Consequently, some staff still needed to develop these competencies [24]. The training needs of staff may be influenced by the nursing home structure, for example whether it is a state or private provider.

Research throughout the pandemic has shown how nursing homes were overlooked and undervalued [1, 2]. Government guidance was slow to address the information needs of nursing home staff with respect to COVID-19 protocols, leaving managers to implement bespoke policies to act in everyone’s best interests. Meanwhile, general information and mis-information about COVID was widespread, creating an ‘infodemic’ which caused further confusion [25]. Professional organisations mobilised, with several producing specific guidance for care staff during this time [4, 26]. Several of these resources helped to inform the intervention [27].

Consequently, there was already a need for nursing home specific information about advance care planning prior to the pandemic, which was exacerbated by COVID-19. Guidance and training produced during the pandemic, including this intervention, demonstrate that care staff and family members require support to undertake advance care planning. Moreover, in some cases, the circumstances of COVID-19 require particular considerations regarding advance care planning which must be articulated to staff and families [4, 26, 27]. Variants of COVID-19 will remain in circulation for some time [28], therefore training and information for nursing homes addressing advance care planning, such as this intervention, will continue to be relevant.

### Theoretical proposition 2: training and information accessibility

While little research exists about the information technology skills of care staff, and ease of computer access in nursing homes, previous research shows that computer-based training is effective for care staff [29]. This project identified similar findings relating to website accessibility and usefulness.

Many studies have been undertaken exploring technology accessibility and online information in relation to family carers [30–33]. Such research often recognises the so-called ‘digital divide’ and technology inequalities that can exist between those who can access health related information online, and those who cannot [32].

Research shows that family carers, particularly older adults, will embrace technology interventions such as online support or information, where such resources are easy to use and provide direct benefits in relation to their caring role [31]. This study reinforces these findings. One study promoting psychological health and coping strategies to carers via online information was viewed positively by users [30]. This is similar to the self-care information provided by this project’s intervention.

However, it is also true that some family carers, at times of care crisis, may feel too overwhelmed or busy to access information online [32]. Furthermore, the quality, relevance and accessibility of online information for carers often varies [33]. It is therefore important not to make any assumptions about accessing information online, regarding technical skills or inclination.

As discussed, residents were not recruited to review the intervention. However, previous research highlights the rights of nursing home residents to access learning opportunities [34]. Future iterations of the intervention should address this discrepancy and be made available to residents where relevant. Moreover, due to financial constraints within this study, it was not possible to offer each family member a computer tablet to ensure they could access the intervention. This policy should be reconsidered in further research to maximise inclusion and prevent unintended technology inequalities.

### Theoretical proposition 3: training and information context

This study demonstrated that organisational and individual contexts were critical in determining the barriers and facilitators regarding engagement with the intervention, a finding that is evident in other research [23, 35–38].

Factors such as leadership buy-in, providing working space and protecting time, are organisational contexts that can significantly influence how effectively training programmes are implemented in nursing homes [35]. The culture of the nursing home can also be important, for example whether continuous professional development is actively promoted [23]. Insufficient resources, namely workforce and finances, are commonly cited as barriers to training care staff [36]. These factors certainly echo findings in this study.

For family members, and residents, a deciding context in accessing information can be timing and readiness to consider advance care planning. Some family members can identify the benefits of proactive and early decision-making, while others may have misconceptions, fears or anxieties [37]. If family members can recognise why advance care planning is needed, they may be more likely to access further information about it. Furthermore, existing family dynamics and relationships with care staff may influence a family member’s willingness to engage with information about advance care planning [38]. Many of these contexts were evident in findings for this study, particularly timing and appetite for information.

### Theoretical proposition 4: encouraging conversations

For care staff, this research showed that improving knowledge and increasing confidence through the training, alongside permission to talk about advance care planning, led to more conversations. Similarly for family members, the research identified that improved knowledge led to feelings of empowerment and a right to be involved, that resulted in greater willingness to engage in conversations about advance care planning.

This link between knowledge and confidence is evident in other research. One study showed that education, alongside other factors such as mentorship, organisational culture and previous experience, boosted care staff confidence to engage in end of life care delivery [39]. Another study, specifically relating to dementia care in care homes, recognised the significance of on-the-job training in enhancing care staff confidence [40].

Permission, a theme identified in the research, is closely related to confidence. Some family members felt that reviewing the intervention had given them permission to ask questions and be involved in their relative’s care. Other research recognises the importance of establishing whether this permission exists in legal terms, and which family members should be consulted on behalf of their loved ones [41].

For some staff members, in homes with strictly delineated roles, the training gave them permission to initiate advance care planning conversations – though this permission needs to be supported by adequate training [42]. Historically, advance care planning may have been viewed as a task for General Practitioners, but increasingly multi-disciplinary professionals are actively initiating these conversations, including care home staff [43].

### Strengths and limitations

This study was a timely response to the COVID-19 pandemic, resulting in a high-quality intervention to support care staff and family members. Recruiting nursing homes from across the UK enabled exploration of a variety of care contexts, which proved critical to the research findings. A significant number of participants and cases enabled rich insights to be drawn from the cross-case analysis.

The case study methodology was limited by COVID-19 restrictions, meaning that it was not possible to visit the case study sites or meet participants. Observation, ethnography and the gathering of tacit insights can be instrumental steps in completing a case study report [9, 10]. However, in some instances, remote interviews via telephone allowed participants to speak more freely than they may have done in face-to-face interviews or within a work environment, resulting in rich data.

This study’s focus on nursing homes could be considered a limitation. However, the participating staff had a combination of nursing and non-nursing backgrounds, suggesting the findings may be applicable across residential homes more broadly. Moreover, while the study focussed on COVID-19, the intervention may have applicability to outbreaks of other infectious diseases or future variants. Further research would help to clarify this.

### Implications for practice and training

The intervention will continue to be available to the homes that have participated in the study. Plans to maintain, update and share the intervention, including with residential homes that do not deliver nursing care, are being considered by the research team. The website can be accessed at: https://covidacpcarehomes.com

From the research findings, it is possible to derive implications for practice, namely recommendations or implementation guidelines relating to care staff training and family member information. These are as follows:

- Consider a consultation period with staff and family members prior to developing resources to clearly identify knowledge gaps and training needs. This enhances the usefulness of the resulting resources and boosts engagement with the materials;
- Ensure training is bite-sized, visually appealing and caters to different learning styles, for example incorporating written and audio-visual materials. Including a quiz offers an opportunity to assess and reflect on learning. Drawing on the diverse perspectives of those with different roles in advance care planning ensures the training and information is relevant and comprehensive;
- Consider the training and information needs of those unable to access online materials;
- The benefits of an online resource may be optimised and reinforced when followed by a facilitated discussion with colleagues, relatives or residents. This gives an opportunity to ask questions and explore how the learning can be applied to the specific context of each nursing home or resident;
- Focussing on the agency of care staff and family members, reinforcing their right to be involved in advance care planning, can help them to feel empowered, valued and confident.

## Conclusion

This research demonstrates that high-quality online training and information for care staff and family members regarding advance care planning during COVID-19 is effective, acceptable and implementable. Providing this training and information can have immediate tangible impacts on staff confidence and family member involvement, leading to more engaged conversations about advance care planning with practical, documented outcomes. It is now important to consider how this intervention can be scaled-up, and made available to more care providers and family members to meet the information and support needs of greater numbers of staff, families and residents.

## Supplementary Information

**Additional file 1. **Graphics taken from the Necessary Discussions training and information website intervention. This file provides images of the following: the website landing page; information for family members; training units for care staff.

## Data Availability

The datasets used and analysed during the current study are available from the corresponding author on reasonable request. The datasets used and analysed during this current study will be uploaded to the ReShare repository: https://reshare.ukdataservice.ac.uk/

## Abbreviations

ERG: Expert Reference Group
RE-AIM: Reach, Effectiveness, Adoption, Implementation, Maintenance framework
TAM: Technology Assessment Model
