# Supplementary material for: Implementing and evaluating online advance care planning training in UK nursing homes during COVID-19: findings from the Necessary Discussions multi-site case study project
Source: BMC Geriatr. 2022 May 13;22:419. doi: 10.1186/s12877-022-03099-z (PMC9098790; doi:10.1186/s12877-022-03099-z)

**Additional files**

**File name**: Additional file 1

**File format**: Microsoft word .docx

**Title of data**: Graphics taken from the Necessary Discussions training and information website intervention

**Description of data**: This file provides images of the following: the website landing page; information for family members; training units for care staff.

**Graphics taken from the Necessary Discussions training and information website intervention**

**The website landing page:**


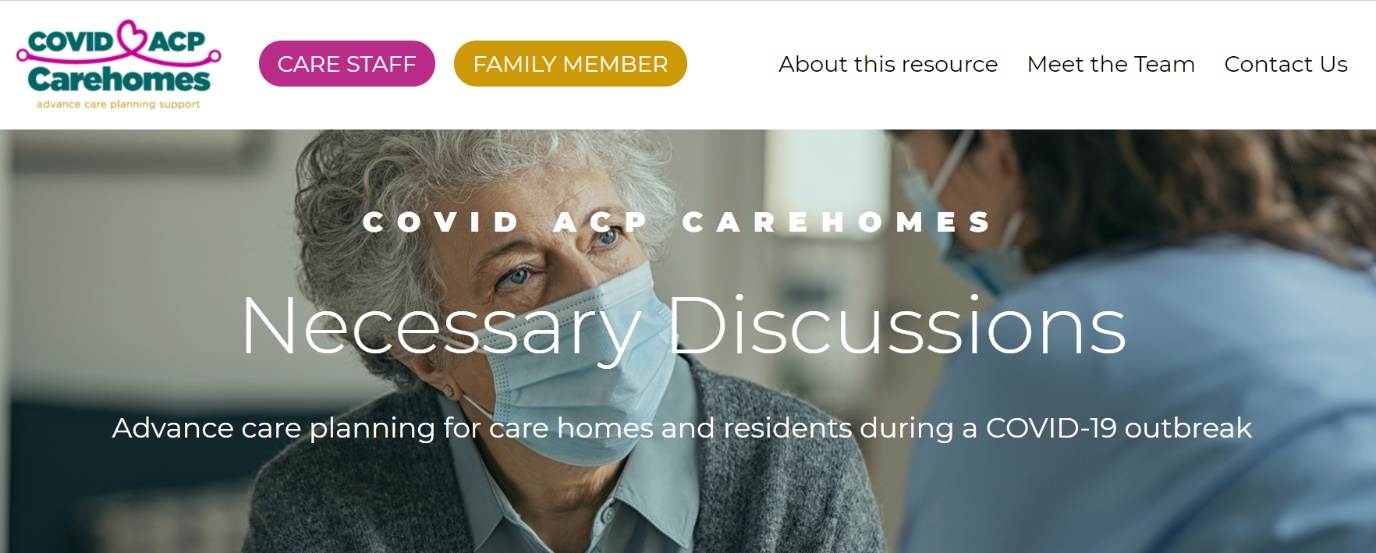


**Information for family members:**


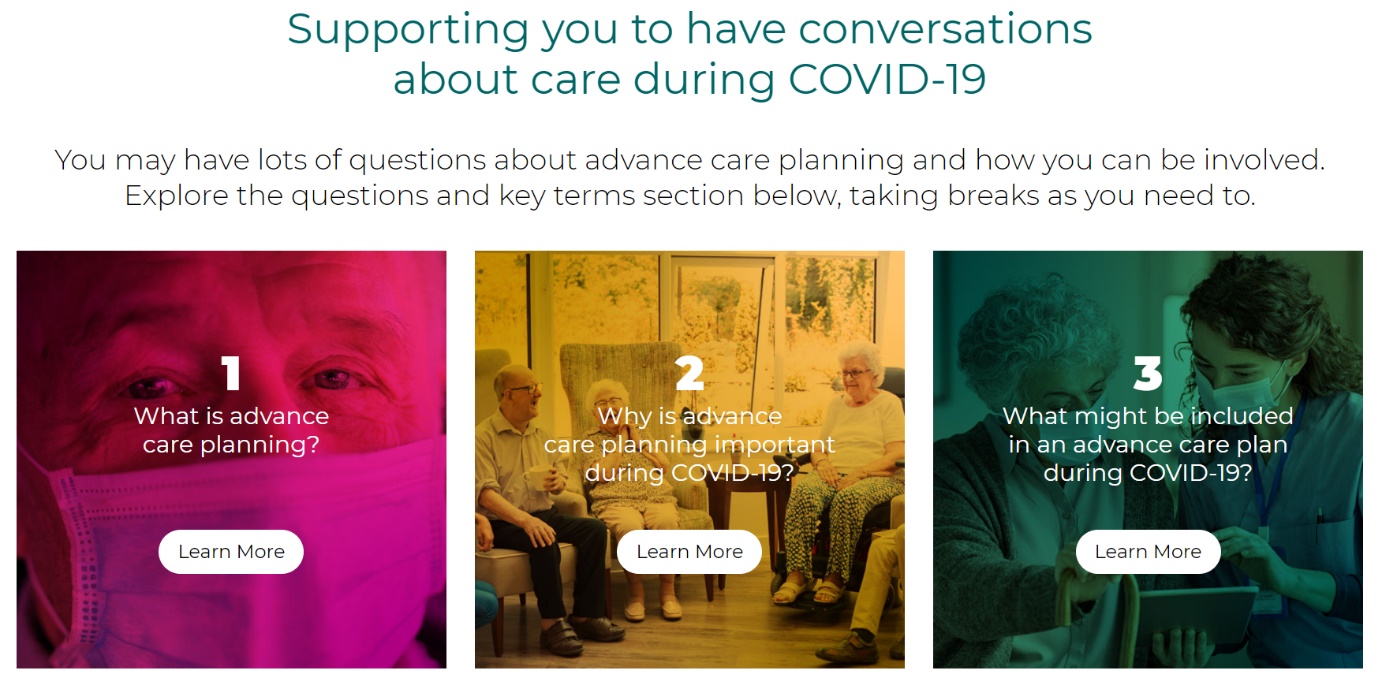


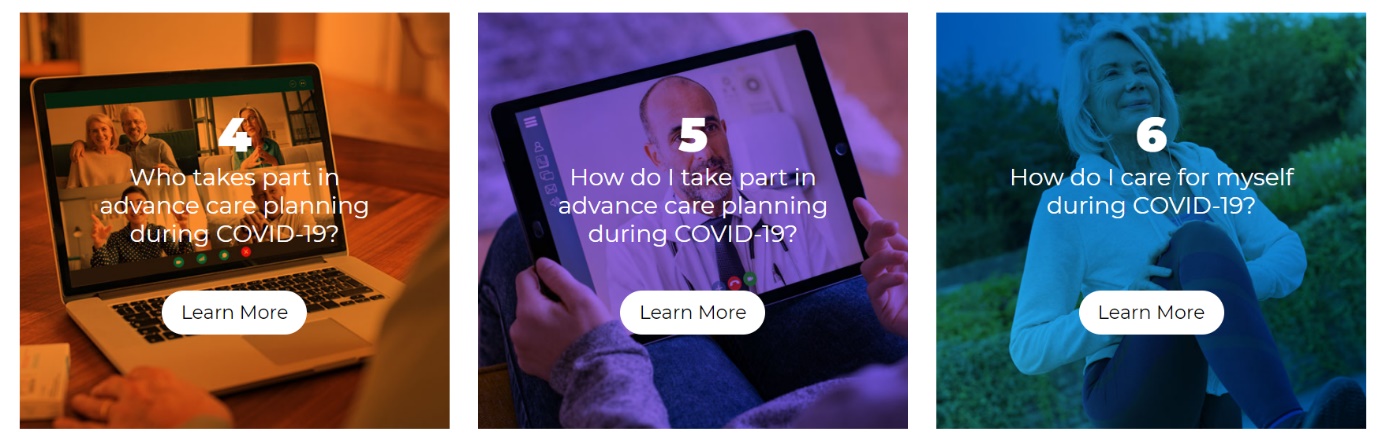


**Training units for care staff:**


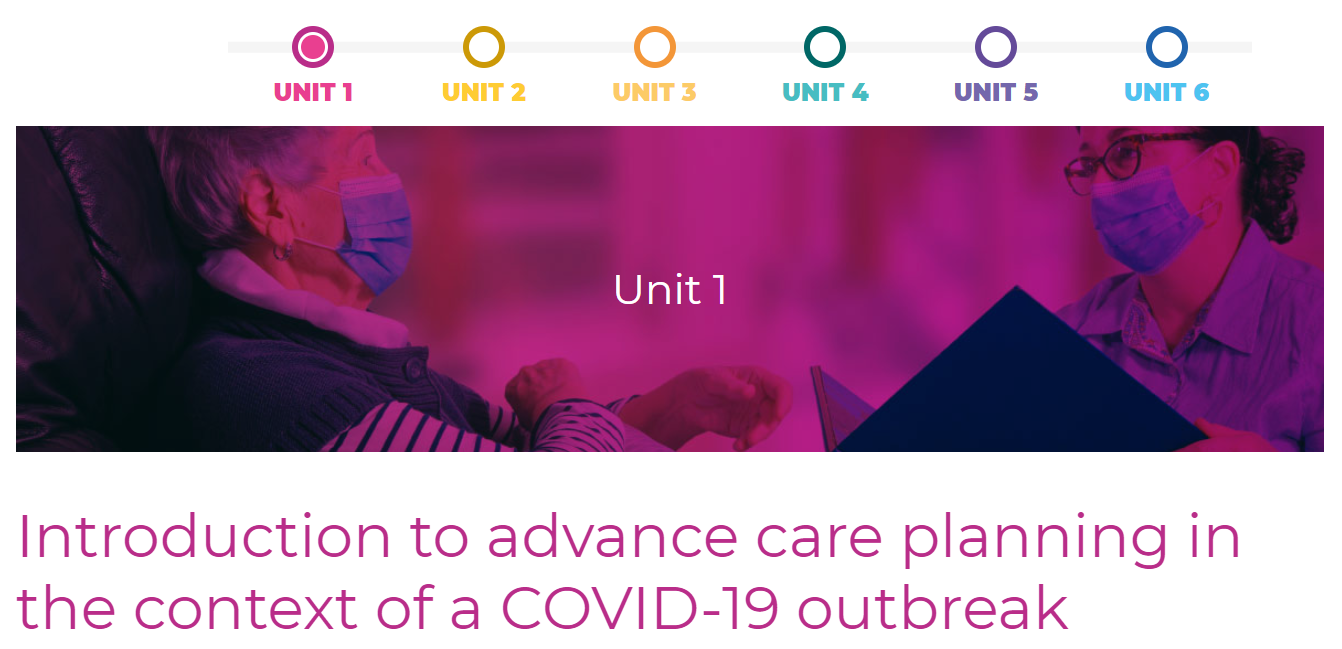


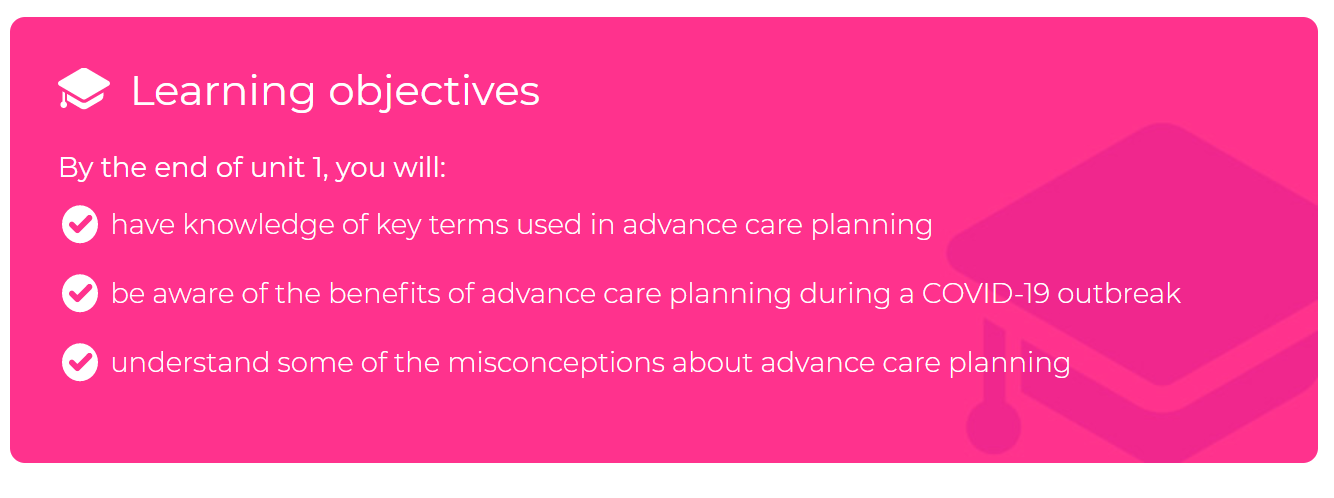

Supplement: Supplementary file 1 — Additional file 1. Graphics taken from the Necessary Discussions training and information website intervention. This file provides images of the following: the website landing page; information for family members; training units for care staff. [file 12877_2022_3099_MOESM1_ESM.docx]
